# Change in cup orientation from supine to standing posture: a prospective cohort study of 419 total hip arthroplasties

**DOI:** 10.2340/17453674.2024.41091

**Published:** 2024-07-22

**Authors:** Camille VORIMORE, Jeroen C F VERHAEGEN, Moritz INNMANN, A Paul MONK, Christopher LING, George GRAMMATOPOULOS

**Affiliations:** 1 Department of Orthopaedic Surgery, The Ottawa Hospital, Ottawa, Ontario, Canada; 2 University Hospital Antwerp, Edegem, Belgium; 3 Orthopaedic Centre Antwerp, AZ Monica, Antwerp, Belgium; 4 Heidelberg University, Heidelberg, Germany; 5 Auckland Surgical Centre, Auckland, New Zealand

## Abstract

**Background and purpose:**

Arthroplasty surgeons traditionally assess cup orientation after total hip arthroplasty (THA) on supine radiographs. Contemporary hip–spine analyses provide information on standing, functional cup orientation. This study aims to (i) characterize cup orientations when supine and standing; (ii) determine orientation differences between postures; and (iii) identify factors associated with magnitude of orientation differences.

**Methods:**

This is a 2-center, multi-surgeon, prospective, consecutive cohort study. 419 primary THAs were included (57% women; mean age: 64 years, standard deviation [SD] 11). All patients underwent supine and standing antero-posterior pelvic and lateral spinopelvic radiographs. Cup orientation and spinopelvic parameters were measured. Target cup orientation was defined as inclination/anteversion of 40°/20° ± 10°. A change in orientation (Δinclination/Δanteversion) between postures > 5° was defined as clinically significant. Variability was defined as 2 x SD.

**Results:**

Inclination increased from 40° (supine) to 42° (standing) corresponding to a Δinclination of 2° (95% confidence interval [CI] 2–3). Anteversion increased from 25° (supine) to 30° (standing) corresponding to a Δanteversion of 5° (CI 5–6). When supine, 69% (CI 65–74) of THAs were within target, but only 44% (CI 39–49) were within target when standing, resulting in a further 26% (CI 21–30) being out of target when standing. From supine to standing, a clinically significant change in anteversion (> 5°) was seen in 47% (CI 42–52) of cases. Δanteversion was higher in women than in men (6°, CI 5–7 vs 5°, CI 4–5) corresponding to a difference of 1° (CI 1–2), which was dependent on tilt change, standing cup anteversion, age, and standing pelvic tilt.

**Conclusion:**

Cup inclination and version increase upon standing but significant variability exists due to patient factors.

Attaining optimal acetabular component positioning is important in total hip arthroplasty (THA) as [1] aberrant cup orientation has been associated with unfavorable outcomes, including impingement [2], dislocation [3], polyethylene wear [4], and patient-reported outcomes [5]. The concept of a “safe” zone has been challenged as researchers have focused on testing whether individual optimum cup orientation targets exist [6].

In recent years, efforts have been focused on understanding how the hip–spine relationship contributes to outcome after THA, and how spinopelvic characteristics can be incorporated into clinical practice and preoperative planning, with a view to improving outcome and reducing dislocation rate [7]. The study of hip–spine interaction is typically performed with quasi-static, standing, and seated radiographs [3,8]. Consequently, the cup orientation recommendations provided are those in the standing position, which satisfy sagittal targets, as per spinopelvic characteristics [3,9].

Surgeons have traditionally assessed cup orientation with supine radiographs and not standing radiographs [10]. The functional cup orientation differs between the standing and supine positions due to changes in pelvic tilt [11]. Each degree of pelvic tilt changes the measurement of radiographic cup anteversion in anteroposterior (AP) radiographs by approximately 0.7° [12]. Cup inclination is also affected by the change in pelvic tilt, but to a lesser degree [13]. On average, the transition from the supine to the standing position is associated with an increase in pelvic tilt (posterior tilt) of approximately 5°; however, the variability is large and factors associated with the degree of change remain to be determined [14].

This study aims to (i) characterize cup orientations when supine and standing, (ii) determine orientation differences between postures, and (iii) identify factors associated with magnitude of orientation differences.

## Methods

### Study design

This is a prospective, multi-surgeon, institutional review board-approved study conducted at 2 tertiary academic institutions (the Ottawa Hospital, Canada and Auckland City Hospital, New Zealand).

All patients who underwent primary THA were recruited. Inclusion criteria for this study were: age older than 16 years and a minimum follow-up of 1 year. None of the surgeries were performed with robotics or navigation

The study is reported according to STROBE guidelines.

### Radiographic assessment

All patients underwent radiographic assessment including supine and standing AP radiographs of the pelvis, in a standardized fashion [15], in line with previous recommendations, at 1 year following THA. In addition, a lateral standing spinopelvic radiograph was conducted preoperatively.

A radiographic analysis of cup orientation was performed with Ein-Bild-Roentgen-Analyse (EBRA-CUP; https://www.ebra.info/ebra-cup-ebragraf/) using AP pelvic radiographs, which has been validated previously [16-18]. For each radiograph, 3 horizontal reference lines were delineated: the first aligned tangentially with the distal aspect of the ischia; the second aligned tangentially with the distal aspect of the teardrops; and the third aligned tangentially with the proximal horizontal border of the greater sciatic notch. Additionally, 3 vertical reference lines were marked: the first at the center of the pubic symphysis; the second at the medial border of each greater sciatic notch, and the third at the lateral aspect of each greater sciatic notch. Following the establishment of these reference lines, a circular outline of the cup component was generated by marking the perimeter of the component with 3 points. Subsequently, an ellipse corresponding to the cup was delineated by marking the vertices of the cup ellipse using 3 additional points. Lateral radiographs were utilized to confirm cup opening in the frontal plane, determining whether it was anteverted or retroverted.

**Figure 1:**
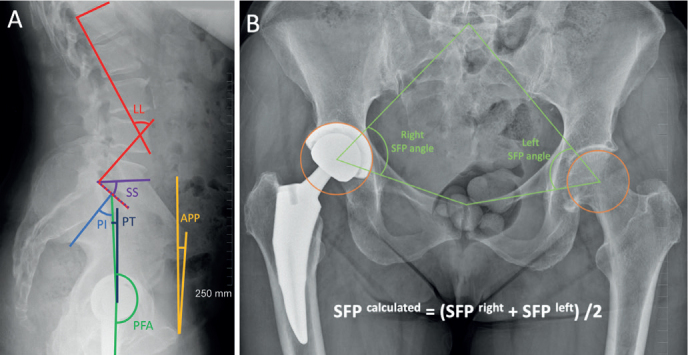
(A) Lateral standing spinopelvic radiographs and (B) AP pelvic radiograph illustrate the measurements performed; LL = Cobb angle between a line drawn along the superior endplate of L1 and another line drawn along the superior endplate of S1; SS = angle between a line drawn along the superior endplate of S1 and the horizontal axis; PI = angle between the line from the center of the cup to the middle of the superior endplate of S1 and the line perpendicular to the superior endplate of S1 from its midpoint; PT = angle formed between the line from the center of the cup to the middle of the superior endplate of S1 and the vertical axis; PFA = angle between the line from the center of the cup to the middle of the superior endplate of S1 and the femoral axis; APP = angle between a line connecting both anterior superior iliac spines with the pubic symphysis and the vertical axis; SFP = angle between a line from the midpoint of the S1 superior endplate (found by determining the midpoint of a line between the lateral bodies of the L5 to S1 facet joints), centroid of the acetabulum, or center of the cup, and upper midpoint of the pubic symphysis.

The following parameters were measured on spinopelvic radiographs (Figure 1): lumbar lordosis (LL), sacral slope (SS), pelvic tilt (PT), pelvic incidence (PI), and pelvic-femoral angle (PFA). The sacro–femoral–pubic angle (SFP) was measured on AP pelvic radiographs. The SFP has been shown to be a reliable parameter to assess the difference in PT in different postures for a given patient [19]. Spinopelvic parameters were missing for 12 patients (no spinopelvic radiograph); there were no other missing values for any of these variables.

All radiographic measurements were performed by 1 observer, a hip surgery research fellow (CV). Measurements were repeated for 10% of randomly selected datasets in a blinded fashion. Intra-observer reliability was calculated using the correlation coefficient with a 2-way mixed model, showing excellent agreement ranging from 0.88 (95% confidence interval [CI] 0.80–0.93) to 0.92 (CI 0.85–0.95).

### Outcomes

Target cup orientation was defined as cup inclination/anteversion of 40°/20° ± 10°, based on values reported in the literature [17,20]. Although these target zones were originally defined on supine radiographs, we chose to investigate the same target zone in the standing position to illustrate how often cups might be in/out of zone according to posture. Previous studies have extended supine targets to standing radiographic analyses [21,22]. Δinclination and Δanteversion were defined as the difference between supine and standing inclination/anteversion (∆inclination = inclination_standing_ – inclination_supine_ and ∆anteversion = anteversion_standing_ – anteversion_supine_). Thresholds of interest with regards to change in orientation (Δinclination/Δanteversion) between postures were defined as > 5° and > 10°, based on previous studies [14]. A difference of > 5° in cup orientations between supine and standing positions was considered to be a clinically important difference [14,21]. Such margins reflect margins of error associated with modern technologies, augmenting ability to achieve target orientation [23]. Variability was defined as 2 x SD.

The change in PT between the supine and standing positions was determined by the difference in the SFP (∆SFP = SFP_supine_ – SFP_standing_), ∆PT was deduced using the ∆SFP angle (∆PT = –∆SFP) [24]. PI–LL mismatch > 10° was defined as an unbalanced spine or “flatback deformity” [25].

Complication and reoperation rates were prospectively recorded.

### Statistics

An a priori power calculation was performed for the primary objective of the study (difference in cup orientation between supine and standing postures) based on the studies of Yun [14]and Tiberi [21] reporting on patients with THA. Cumulatively, Yun et al. reported an increase in the mean (standard deviation [SD]) cup inclination from supine 44° (SD 5) to standing 45° (SD 5), and in cup anteversion from supine 26° (SD 10) to standing 29° (SD 8). Thus, at least 336 hips would be required to provide sufficient study power (1–β = 0.80, α = 0.05).

Means and change of inclination and anteversion between postures were compared with paired t-tests. To compare continuous variables between independent groups, a Mann–Whitney U test was used. A chi-square exact test was used to test for differences between categorical variables. The results were presented as mean (CI) for continuous variables, and for categorical data, they were presented as absolute count (percentages). Pearson’s correlation coefficient rho (ρ) was calculated to compare individual anatomical parameters with acetabular morphology variations, and graded as poor (ρ ≤ 0.30), moderate (ρ 0.31–0.50), or strong (ρ > 0.51). Statistical analysis was performed using SPSS v25 (IBM Corp, Armonk, NY, USA). A P value < 0.05 was considered significant.

### Ethics, registration, data sharing, use of AI, funding, and disclosures

The study received approval from the Institutional Review Board (IRB). Restrictions apply to the availability of this data, which was used under license for the current study. All participants signed written informed consent. No artificial intelligence was used in this study. No benefits in any form have been received or will be received related directly or indirectly to the subject of this article. Authors report no conflict of interests. Completed disclosure forms for this article following the ICMJE template are available on the article page, doi: 10.2340/17453674.2024.41091

## Results

### Cohort description

**Figure 2:**
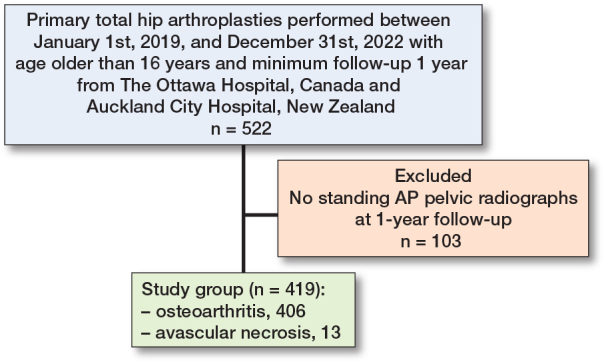
Number of patients included in analysis and reasons for loss to follow-up

522 hips underwent primary THA at the 2 centers between January 1, 2019, and December 31, 2022, by 11 surgeons. 103 without standing AP pelvic radiographs at 1-year follow-up were excluded, leaving 419 THAs (patients) eligible for inclusion (Figure 2). There were 180 (43%) men and 239 (57%) women. The mean age was 64 (SD 11) years, and the mean BMI was 28 (SD 5).

Indication for THA was osteoarthritis in 406 (97%), or avascular necrosis in 13 (3%). THA was mostly performed through an anterior (56%), followed by a posterior (38%) and a lateral (6%) approach (Table 1). Approach and implant choice was as per individual surgeon preference and institutional inventory. The most common femoral head diameter was 36 mm. Most stem implants and all cup implants were cementless (Table 1). However, an intraoperative radiograph to assess the hip was performed prior to closure in 264 (63%) as per surgeon preference.

**Table 1:** Demographics and surgical details of the study cohort. Values are count (%) unless otherwise specified

Factor	Cohort (n = 419)
Age, mean (SD)	64 (11)
Sex	
Women	239 (57)
Men	180 (43)
BMI (SD)	28 (5)
Indication for primary THA	
Osteoarthritis	406 (97)
AVN	13 (3.1)
Approach	
Anterior	234 (56)
Posterior	158 (38)
Lateral	27 (6.4)
Femoral head size, mm	
28	17 (4.1)
32	103 (25)
36	245 (58)
Others	54 (13)
Femoral implant	
Microplasty	214 (51)
Taperloc	86 (21)
Exeter	43 (10)
Sirius	19 (4.5)
Corail	11 (2.6)
Others	46 (11)
Cup implant	
G7	312 (74)
Pinnacle	5 (1.2)
Others	102 (24)
Stem fixation	
Uncemented	365 (87)
Cemented	54 (13)
Complications	
Fracture	10 (2.3)
Prosthetic joint infection	6 (1.4)
Dislocation	1 (<1)
Reoperations for	
Fracture	9 (2.1)
Prosthetic joint infection	6 (1.4)
Dislocation	1 (< 1)

The overall complication rate was 17 (4%). There were 10 periprosthetic femoral fractures (2%), 6 prosthetic joint infections (PJI) (1%), and 1 dislocation (< 1%). 16 (4%) patients required reoperation: 9 peri-prosthetic fracture (2%), 6 PJI (1%), and 1 dislocation (< 1%) (Table 1).

### Cup orientation in supine and standing position

**Figure 3:**
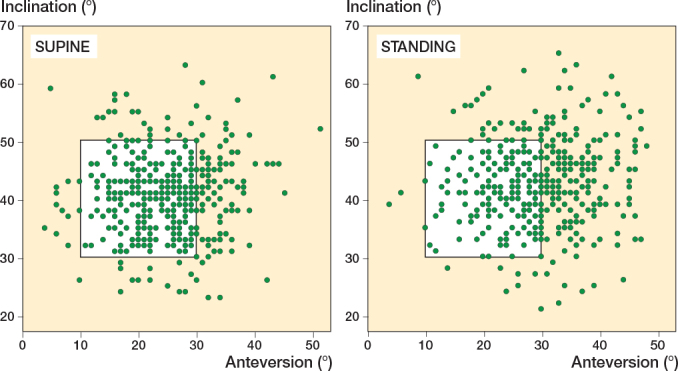
Cup inclination and anteversion in supine and standing. The percentage of cups within the supine safe zone (white area) was greater in supine than in standing. In the standing position, half of the cups exhibited anteversion higher than the safe zone.

Mean cup inclination was 40° (SD 7; CI 40–41) supine and 43° (SD 7; CI 42–43) standing. Similarly, mean cup anteversion was 25° (SD 7; CI 24–25) supine and 30° (SD 8; CI 29–31) standing. Supine, 290 (69%, CI 65–74) of THAs were within target cup orientation (anteversion and inclination), and 183 of cups (44%, CI 39–49) were within target when standing, resulting in a further 107 (26%, CI 21–30) being out of target when standing (Table 2 and Figure 3).

**Table 2:** Radiographic parameters in supine and standing positions (N = 419). Continuous values are mean [CI], categorical values are number (%) [CI of %)]

Factor	Supine	Standing	∆	P value
SFP (°)	61 [60–62]	56 [56–56.9]	–5 [4–5]	< 0.001 **^a^**
Cup inclination (°)	40 [40–41]	43 [42–43]	2 [2–3]	< 0.001 **^a^**
Cup inclination amongst approaches (°)				0.7 **^a^**
anterior approach	41 [40–41]	43 [42–44]	2 [0–2]	
posterior approach	40 [39–42]	43 [42–44]	2 [2–3]	
lateral approach	40 [37–43]	43 [39–46]	3 [1–4]	
Cup anteversion (°)	25 [24–25]	30 [29–31]	5 [5–6]	< 0.001 **^a^**
Cup anteversion amongst approaches (°)				0.5 **^a^**
anterior approach	25 [24–26]	30 [29–31]	6 [5–6]	
posterior approach	25 [23–26]	30 [28–31]	5 [4–6]	
lateral approach	22 [18–25]	27 [23–32]	5 [3–8]	
Inclination in the target	369 (88) [84–91]	348 (83) [79–87]	–21 (5) [3–7]	< 0.001 **^b^**
Anteversion in the target	325 (78) [73–82]	213 (51) [46–56]	–112 (27) [23–30]	< 0.001 **^b^**
Inclination and anteversion in the target	290 (69) [65–74]	183 (44) [39–49]	–107 (26) [21–30]	< 0.001 **^b^**
LL (°)		53 [52–55]		
SS (°)		38 [38–39]		
PI (°)		54 [53–56]		
PT (°)		16 [15–17]		
PFA (°)		191 [190–192]		
PI–LL (°)		1 [0–2]		
PI–LL >10°		89 (21) [17–25]		
LL <45°		103 (25) [21–30]		

∆ = difference between standing and supine; SFP = sacro–femoral–pubic angle; LL = lumbar lordosis; SS = sacral slope; PI = pelvic incidence; PT = pelvic tilt; PFA = the pelvic-femoral angle.

The target = 40° ± 10° in inclination and 20° ± 10° in anteversion.

a Paired samples t-tests.

b Chi-square.

No differences between approaches were seen (Table 2). The 1 hip with instability had an anteversion of 20° and an inclination of 43° in the supine position. There was a 3° change in anteversion and a 2° change in inclination between the supine and standing positions.

### Cup orientation differences between postures

Δinclination increased by 2° (SD 3, CI 2–3) and Δanteversion by 5° (SD 4, CI 5–6) between positions (Table 2 and Figure 3). When transitioning from supine to standing, 196 (47%, CI 42–52) exhibited changes in anteversion of 5° or more, while 49 (12%, CI 9–15) showed changes in inclination of 5° or more (Table 3).

**Table 3:** Cup anteversion variation between supine and standing in subgroups. Continuous values are mean [CI], categorical values are number (%) [CI of %]

Factor	< 5°	≥ 5°	∆ anteversion P value	< 10°	≥ 10°	P value
Cases	223 (53) [48–58]	196 (47) [42–52]		365 (87) [84–90]	54 (13) [10–16]	
Age, mean	64 [62–65]	65 [64–67]	0.2 a	64 [63–65]	67 [65–70]	0.053 **^a^**
Sex			0.02 **^b^**			< 0.001 **^b^**
Women	115 (52) [45–58]	124 (63) [56–70]		195 (53) [48–59]	44 (81) [69–91]	
Men	108 (48) [42–55]	72 (37) [30–44]		170 (47) [42–52]	10 (19) [9–31]	
Anteversion supine (°)	25 [24–26]	24 [23–25]	0.3 **^a^**	25 [24–25]	25 [23–26]	0.9 **^a^**
Anteversion standing (°)	27 [26–28]	33 [32–34]	< 0.001 **^a^**	29 [28–30]	37 [35–39]	< 0.001 **^a^**
∆SFP (°)	3 [3–4]	7 [6–7]	< 0.001 **^a^**	4 [4–4]	9 [8–10]	< 0.001 **^a^**
PI standing (°)	53 [52–55]	56 [54–57]	0.06 **^a^**	54 [53–56]	54 [51–58]	1 **^a^**
PT standing (°)	15 [14–16]	17.5 [16–19]	0.002 **^a^**	16 [15–17]	19 [17–22]	0.003 **^a^**
PFA standing (°)	190 [189–191]	193 [191–194]	0.007 **^a^**	191 [190–192]	193 [191–196]	0.08 **^a^**
PI–LL > 10°	46 (21) [16–27]	43 (23) [17–29]	0.8 **^b^**	79 (17) [13–22]	10 (19) [10–33]	0.6 **^b^**
LL < 45°	63 (29) [23–36]	40 (21) [15–27]	0.057 **^b^**	91(26) [21–31]	12 (23) [13–37]	0.7 **^b^**

For Abbreviations, see Table 2.

a Mann–Whitney test.

b Chi-square.

### Factors associated with magnitude of orientation difference

Δanteversion showed weak correlation with standing PT (ρ 0.22, CI 0.13–0.31), moderate correlation with standing cup anteversion (ρ 0.48, CI 0.40–0.55), and strong correlation with ∆SFP (ρ 0.62, CI 0.55–0.67). Similarly, ∆inclination exhibited weak correlation with standing PT (ρ 0.13, CI 0.03–0.23) and standing PFA (ρ 0.20, CI 0.10–0.29), moderate correlation with standing cup anteversion (ρ 0.29, CI 0.20–0.38) and inclination (ρ 0.37, CI 0.28–0.45), and strong correlation with ∆SFP (ρ 0.55, CI 0.48–0.62) (Tables 3 and 4).

**Table 4:** Cup orientation variations in supine and standing between individual parameters. Values are mean [CI]

Factor	∆inclination (°)	P value	∆anteversion (°)	P value
Sex		0.009 **^a^**		0.006 **^a^**
women (n = 239)	3 [2 to 3]		6 [5 to 7]	
men (n = 180)	2 [1 to 2]		5 [4 to 5]	
Approach		0.5 **^b^**		0.5 **^b^**
anterior (n = 234)	2 [2 to 3]		6 [5 to 6]	
posterior (n = 158)	2 [2 to 3]		5 [4 to 6]	
lateral (n = 27)	3 [1 to 5]		6 [4 to 9]	
Dislocation		1 **^a^**		0.5 **^a^**
yes (n = 1)	2		3	
no (n = 418)	2 [2 to 3]		5 [5 to 6)	
Age, rho	0.08 [–0.02 to 0.18]	0.1 **^c^**	0.10 [0.01 to 0.20]	0.04 **^c^**
BMI, rho	0.04 [–0.07 to 0.14]	0.5 **^c^**	–0.04 [–0.14 to 0.07]	0.5 **^c^**
Inclination supine, rho	0.02 [–0.08 to 0.11]	0.7 **^c^**	–0.03 [–0.13 to 0.06]	0.5 **^c^**
Anteversion supine, rho	0.14 [0.04 to 0.23]	0.006 **^c^**	–0.04 [–0.14 to 0.05]	0.4 **^c^**
Inclination standing, rho	0.37 [0.28 to 0.45]	< 0.001 **^c^**	0.09 [–0.01 to 0.18]	0.08 c
Anteversion standing, rho	0.29 [0.20 to 0.38]	< 0.001 **^c^**	0.48 [0.40 to 0.55]	< 0.001 **^c^**
∆SFP, rho	0.55 [0.48 to 0.62]	< 0.001 **^c^**	0.62 [0.55 to 0.67]	< 0.001 **^c^**
LL standing, rho	0.05 [–0.04 to 0.15]	0.3 **^c^**	0.07 [–0.03 to 0.17]	0.2 **^c^**
SS standing, rho	–0.07 [–0.17 to 0.03]	0.1 **^c^**	–0.09 [–0.18 to 0.01]	0.08 **^c^**
PI standing,rho	0.03 [–0.07 to 0.3]	0.6 **^c^**	0.10 [0.01 to 0.19]	0.049 **^c^**
PT standing, rho	0.13 [0.03 to 0.23]	0.009 **^c^**	0.22 [0.13 to 0.31]	< 0.001 **^c^**
PFA standing, rho	0.20 [0.10 to 0.29]	< 0.001 **^c^**	0.17 [0.07 to 0.26]	0.001 **^c^**
PI–LL standing, rho	–0.03 [–0.13 to 0.07]	0.6 **^c^**	0.01 [–0.08 to 0.11]	0.8 **^c^**
∆Leg length, rho	–0.05 [–0.16 to 0.06]	0.4 **^c^**	–0.08 [–0.19 to –0.03]	0.2 **^c^**

For Abbreviations, see Table 2.

a Mann–Whitney.

b Kruskal–Wallis.

c Pearson’s correlation.

Women exhibited greater Δinclination (3°, CI 2–3 vs 2°, CI 1–2) and Δanteversion (6°, CI 5–7 vs 5°, CI 4–5) than men, corresponding to a difference of 1° (CI 0–1) in ∆inclination and 1° (CI 1–2) in ∆anteversion. Women were more likely to exhibit Δanteversion greater than 5°, and also 10° (Table 3), had greater ∆SFP (5°, CI 5–6 vs 4°, CI 4–5) and greater supine (26°, CI 25–27 vs 23°, CI 22–24) and standing anteversion (32°, CI 31–33 vs 27°, CI 26–28) than men.

Patients with reduced lumbar lordosis (LL < 45°) or flatback deformity (PI–LL > 10°) did not exhibit a difference in cup anteversion variation between the supine and standing positions compared with the rest of the cohort.

## Discussion

This study aims to (i) characterize cup orientations when supine and standing; (ii) determine orientation differences between postures; and (iii) identify factors associated with magnitude of orientation differences. We showed that the percentage of patients with cup orientation within target range was higher in the supine position than when standing, which is unsurprising as such targets have been set with studies analyzing supine radiographic cup data [5,20]. Furthermore, transitioning from supine to standing leads to an increase in cup inclination and anteversion, which would lead to many cups considered to be mal-orientated due to high cup inclination and/or version. Consequently, planning for a standing cup orientation using values established from supine assessments would lead to a substantial reduction in anteversion and inclination when supine. Many cups with orientations of 40 ± 10°/20 ± 10° for inclination/anteversion when standing would exhibit low cup version when supine. Thus, standing cup orientation boundaries need to be redefined if standing cup orientations are to be used as the orientation of relevance in hip arthroplasty. In our study, the main factors that could predict the change in acetabular anteversion between the two positions that should be considered during component implantation were the change in pelvic tilt between positions, standing pelvic tilt, and the amount of acetabular inclination/anteversion.

Transitioning from supine to standing is associated with an increase in inclination and anteversion of approximately 2° and 5° respectively. This is similar to previous literature reporting a change in inclination of 3° and a change in anteversion of 6° between supine and standing [14]. Similarly, Tiberi et al. reported a prevalence of 53% of patients presenting a variation of more than 5° in cup anteversion/inclination between supine and standing position [21]. The number of patients with cup orientation within the target zone of 40° ± 10° anteversion and 20° ± 10° inclination was higher in the supine position (69%) compared with the standing position (44%). Several studies have questioned the effectiveness of a so-called “safe” zone to predict dislocation risk [10]. However, failure to appreciate differences in cup orientation between postures may lead to cup orientation being erroneously mislabeled as “non-optimal” or “optimal” in the literature. Our study demonstrated that utilizing the zonal boundaries defined on supine patient positions may not be applicable to standing patient positions due to the differences in cup orientation between supine and standing positions. Therefore, a distinct optimum zone for cup orientation should be established for standing positions. Thus, more studies with standing position measurements should assess which functional orientation (supine or standing) is superior in identifying at-risk patients for dislocation and what cup orientation margins are acceptable.

When standing the hip is extended and at potential risk of posterior-impingement and anterior dislocation (especially with further femoral extension and external rotation)[3]. A significant increase in cup inclination and anteversion when standing is associated with a reduction in space associated with implant-on-implant impingement posteriorly. Factors that further exacerbate posterior impingement risk are increased femoral version and reduced offset (more common in females). Individual assessment of how tilt changes between supine and standing postures is thus of value and should be accounted for as a significant change in orientation between postures was seen in many cases but not consistently for all [7]. Variability (2 x SD) among individuals was 5° for a difference in inclination and 9° for a difference in anteversion between postures, and 47% exhibited Δanteversion of 5° or more. Certain demographic and anatomical variables were associated with changes in cup orientation between postures [26]. 80% of patients with a difference in anteversion > 10° between supine and standing were women. Women exhibited greater change in pelvic tilt (ΔSFP) and greater cup anteversion, both factors contributing to Δanteversion seen between supine and standing. Women exhibit greater pelvic mobility compared with men when transitioning between postures [27]. Furthermore, we found that age was correlated with an increase in anteversion. An age-related increase in PT variation between supine and standing was previously observed by Hamada et al., who found a variability of 5° preoperatively compared with 10° variability at 20 years postoperatively [28]. Such findings and observations would mean that increased caution is necessary not to result with excessive functional cup anteversion in all patients and especially in older females with degenerative spines as they may have compound risk for posterior impingement due to morphological factors (offset, version, tilt).

In our study, change in pelvic tilt between supine and standing—as measured by ∆SFP—was 5°, which is in line with previous studies among patients treated with THA [14,29]. The degree of change in pelvic tilt was strongly correlated with changes in both inclination and anteversion. The variation in cup orientation is therefore secondary to an increase in posterior pelvic tilt occurring upon standing. While the difference in anteversion between supine and standing positions showed a weak correlation with standing pelvic tilt, PTstanding was still 4° higher in cups that demonstrated a difference in anteversion of more than 10°. Patients with a higher PTstanding may therefore be expected to have a bigger difference in cup anteversion between supine and standing [21].

### Limitations

First, cup orientation measurements were conducted using supine anteroposterior pelvic radiographs. Use of radiographs is sensitive to the center of the beam and can only account for pelvic obliquity, therefore not considering pelvic rotation or tilt, both of which affect cup orientation. The utilization of supine and standing CT scans would have enhanced the precision and accuracy of cup orientation measurements. However, it was important to perform a pragmatic study that would be applicable as per standard of care provided in both centers without subjecting the patients to increased radiation exposure. Second, the outcomes derived from EBRA depend on the operator and the predetermined reference points, which may lead to measurement inaccuracies, especially when multiple cup designs are used. However, previous studies have demonstrated the validity and reproducibility of EBRA in conducting these specific measurements [30]. Additionally, as the objective of the study was to compare cup orientation between the supine and standing positions, the emphasis was not on providing absolute measurement values but rather on highlighting differences. As a result, the risk of bias in measurement errors in cup orientation was minimized. Third, it was decided not to obtain lateral spinopelvic radiographs when supine, to minimize the cohort’s radiation exposure. For assessment of the change, SFP has been shown to have an excellent ability to detect changes in pelvic tilt and was thus considered the parameter of choice [19]. Fourth, as there was only 1 dislocation in the cohort, we were unable to identify a specific cup positioning zone associated with a lower risk of dislocation. However, the primary aim of the study was to quantify the change in anteversion and inclination between supine and standing positions. How supine and standing cup orientations vary between unstable and stable THAs has been reported previously [3].

### Conclusion

Cup orientation differs significantly between supine and standing postures. Upon standing, both cup inclination and version increase. The variability of change is about 5° for inclination and 9° for anteversion. Women, advanced age, patients with a high cup anteversion, and a high standing pelvic tilt exhibit greater changes in cup orientation. This should be considered during preoperative planning; as surgeons position the cup in the supine position, these patients are at risk of presenting with excessive anteversion in the standing position.

In perspective, standing cup orientation boundaries need to be redefined if standing cup orientations are to be used when accounting for spinopelvic characteristics preoperatively.

## Notes

CV and GG were involved in acquisition, analysis, and interpretation of the data and in drafting the manuscript. JCFV, MI, APM, CL. and OAG were involved in interpretation of the data and in drafting the manuscript.

Handling co-editors: Marianne Westberg and Robin Christensen

*Acta* thanks Arild Aamodt and Bart L Kaptein for help with peer review of this manuscript.

## References

[1] Meermans G, Grammatopoulos G, Innmann M, Beverland D. Cup placement in primary total hip arthroplasty: how to get it right without navigation or robotics. EFORT Open Rev 2022; 7(6): 365-74. doi: 10.1530/EOR-22-0025. 35638598 PMC9257731

[2] Jolles B M, Zangger P, Leyvraz P-F. Factors predisposing to dislocation after primary total hip arthroplasty: a multivariate analysis. J Arthroplasty 2002; 17(3): 282-8. doi: 10.1054/arth.2002.30286. 11938502

[3] Grammatopoulos G, Falsetto A, Sanders E, Weishorn J, Gill H S, Beaulé P E, et al. Integrating the combined sagittal index reduces the risk of dislocation following total hip replacement. J Bone Joint Surg Am 2022; 104(5): 397-411. doi: 10.2106/JBJS.21.00432. 34767540

[4] Sculco P K, Austin M S, Lavernia C J, Rosenberg A G, Sierra R J. Preventing leg length discrepancy and instability after total hip arthroplasty. Instr Course Lect 2016; 65: 225-41. 27049193

[5] Grammatopoulos G, Thomas G E R, Pandit H, Beard D J, Gill H S, Murray D W. The effect of orientation of the acetabular component on outcome following total hip arthroplasty with small diameter hard-on-soft bearings. Bone Joint J 2015; 97-B(2): 164-72. doi: 10.1302/0301-620X.97B2.34294. 25628277

[6] Innmann M M, Maier M W, Streit M R, Grammatopoulos G, Bruckner T, Gotterbarm T, et al. Additive influence of hip offset and leg length reconstruction on postoperative improvement in clinical outcome after total hip arthroplasty. J Arthroplasty 2018; 33(1): 156-61. doi: 10.1016/j.arth.2017.08.007. 28887022

[7] Grammatopoulos G, Innmann M, Phan P, Bodner R, Meermans G. Spinopelvic challenges in primary total hip arthroplasty. EFORT Open Rev 2023; 8(5): 298-312. doi: 10.1530/EOR-23-0049. 37158334 PMC10233804

[8] Bodner R J. The functional mechanics of the acetabular component in total hip arthroplasty. J Arthroplasty 2022; 37(11): 2199-2207.e1. doi: 10.1016/j.arth.2022.05.017. 35643259

[9] Vigdorchik J M, Sharma A K, Buckland A J, Elbuluk A M, Eftekhary N, Mayman D J, et al. 2021 Otto Aufranc Award: A simple hip–spine classification for total hip arthroplasty: validation and a large multicentre series. Bone Joint J 2021; 103-B(7 Supple B): 17-24. doi: 10.1302/0301-620X.103B7.BJJ-2020-2448.R2. 34192913

[10] Abdel M P, von Roth P, Jennings M T, Hanssen A D, Pagnano M W. What safe zone? The vast majority of dislocated THAs are within the Lewinnek safe zone for acetabular component position. Clin Orthop Relat Res 2016; 474(2): 386-91. doi: 10.1007/s11999-015-4432-5. 26150264 PMC4709312

[11] Lazennec J-Y, Charlot N, Gorin M, Roger B, Arafati N, Bissery A, et al. Hip–spine relationship: a radio-anatomical study for optimization in acetabular cup positioning. Surg Radiol Anat 2004; 26(2): 136-44. doi: 10.1007/s00276-003-0195-x. 14605752

[12] Lembeck B, Mueller O, Reize P, Wuelker N. Pelvic tilt makes acetabular cup navigation inaccurate. Acta Orthop 2005; 76(4): 517-23. doi: 10.1080/17453670510041501. 16195068

[13] Ranawat C S, Ranawat A S, Lipman J D, White P B, Meftah M. Effect of spinal deformity on pelvic orientation from standing to sitting position. J Arthroplasty 2016; 31(6): 122-27. doi: 10.1016/j.arth.2015.11.035. 26725131

[14] Yun H, Murphy W S, Ward D M, Zheng G, Hayden B L, Murphy S B. Effect of pelvic tilt and rotation on cup orientation in both supine and standing positions. J Arthroplasty 2018; 33(5): 144-28. doi: 10.1016/j.arth.2017.11.069. 29276116

[15] Clohisy J C, Carlisle J C, Beaulé P E, Kim Y-J, Trousdale R T, Sierra R J, et al. A systematic approach to the plain radiographic evaluation of the young adult hip. J Bone Joint Surg Am 2008; 90(Suppl 4): 47-66. doi: 10.2106/JBJS.H.00756. PMC2682767 18984718

[16] Stoeckl B, Biedermann R, Auckenthaler T, Bach C, Sununu T, Nogler M. Ante- and retroversion measurements of cups by EBRA. J Bone Joint Surg Br 2001; 83.11245544

[17] Grammatopoulos G, Pandit H, Glyn-Jones S, McLardy-Smith P, Gundle R, Whitwell D, et al. Optimal acetabular orientation for hip resurfacing. J Bone Joint Surg Br 2010; 92(8): 1072-8. doi: 10.1302/0301-620X.92B8.24194. 20675749

[18] Langton D J, Sprowson A P, Mahadeva D, Bhatnagar S, Holland J P, Nargol A V F. Cup anteversion in hip resurfacing: validation of EBRA and the presentation of a simple clinical grading system. J Arthroplasty 2010; 25(4): 607-13. doi: 10.1016/j.arth.2009.08.020. 20022454

[19] Innmann M M, McGoldrick N P, Ratra A, Merle C, Grammatopoulos G. The accuracy in determining pelvic tilt from anteroposterior pelvic radiographs in patients awaiting hip arthroplasty. J Orthop Res 2022; 40(4): 854-61. doi: 10.1002/jor.25115. 34081347

[20] Lewinnek G E, Lewis J L, Tarr R, Compere C L, Zimmerman J R. Dislocations after total hip-replacement arthroplasties. J Bone Joint Surg Am 1978; 60(2): 217-20. 641088

[21] Tiberi J V, Antoci V, Malchau H, Rubash H E, Freiberg A A, Kwon Y-M. What is the fate of total hip arthroplasty (THA) acetabular component orientation when evaluated in the standing position? J Arthroplasty 2015; 30(9): 1555-60. doi: 10.1016/j.arth.2015.03.025. 25863890

[22] Teeter M G, Goyal P, Yuan X, Howard J L, Lanting B A. Change in acetabular cup orientation from supine to standing position and its effect on wear of highly crosslinked polyethylene. J Arthroplasty 2018; 33(1): 26-37. doi: 10.1016/j.arth.2017.08.016. 28917617

[23] Ando W, Takao M, Hamada H, Uemura K, Sugano N. Comparison of the accuracy of the cup position and orientation in total hip arthroplasty for osteoarthritis secondary to developmental dysplasia of the hip between the Mako robotic arm-assisted system and computed tomography-based navigation. Int Orthop 2021; 45(7): 1719-25. doi: 10.1007/s00264-021-05015-3. 33880612

[24] Blondel B, Schwab F, Patel A, Demakakos J, Moal B, Farcy J-P, et al. Sacro-femoral-pubic angle: a coronal parameter to estimate pelvic tilt. Eur Spine J 2012; 21(4): 719-24. doi: 10.1007/s00586-011-2061-6. 22113529 PMC3326127

[25] Buckland A J, Fernandez L, Shimmin A J, Bare J V, McMahon S J, Vigdorchik J M. Effects of sagittal spinal alignment on postural pelvic mobility in total hip arthroplasty candidates. J Arthroplasty 2019; 34(11): 2663-8. doi: 10.1016/j.arth.2019.06.036. 31301908

[26] Verhaegen J C F, Innmann M, Alves Batista N, Dion C-A, Horton I, Pierrepont J, et al. Defining “normal” static and dynamic spinopelvic characteristics: a cross-sectional study. JBJS Open Access 2022; 7(3): e22.00007. doi: 10.2106/JBJS.OA.22.00007. PMC9260734 35812809

[27] Shareghi B, Mohaddes M, Kärrholm J. Pelvic tilt between supine and standing after total hip arthroplasty an RSA up to seven years after the operation. J Orthop Res 2021; 39(1): 12-19. doi: 10.1002/jor.24759. 32484957

[28] Hamada H, Uemura K, Takashima K, Ando W, Takao M, Sugano N. What changes in pelvic sagittal tilt occur 20 years after THA? Clin Orthop Relat Res 2023; 481(4): 690-9. doi: 10.1097/CORR.0000000000002382 36040725 PMC10013667

[29] Pierrepont J W, Feyen H, Miles B P, Young D A, Baré J V, Shimmin A J. Functional orientation of the acetabular component in ceramic-on-ceramic total hip arthroplasty and its relevance to squeaking. Bone Joint J 2016; 98-B(7): 910-6. doi: 10.1302/0301-620X.98B7.37062. 27365468

[30] Ilchmann T, Kesteris U, Wingstrand H. EBRA improves the accuracy of radiographic analysis of acetabular cup migration. Acta Orthop Scand 1998; 69(2): 119-24. doi: 10.3109/17453679809117610. 9602766

